# Individual sequences in large sets of gene sequences may be distinguished efficiently by combinations of shared sub-sequences

**DOI:** 10.1186/1471-2105-6-90

**Published:** 2005-04-08

**Authors:** Mark J Gibbs, John S Armstrong, Adrian J Gibbs

**Affiliations:** 1 School of Botany and Zoology, Faculty of Science, Australian National University, ACT 0200, Australia

## Abstract

**Background:**

Most current DNA diagnostic tests for identifying organisms use specific oligonucleotide probes that are complementary in sequence to, and hence only hybridise with the DNA of one target species. By contrast, in traditional taxonomy, specimens are usually identified by 'dichotomous keys' that use combinations of characters shared by different members of the target set. Using one specific character for each target is the least efficient strategy for identification. Using combinations of shared bisectionally-distributed characters is much more efficient, and this strategy is most efficient when they separate the targets in a progressively binary way.

**Results:**

We have developed a practical method for finding minimal sets of sub-sequences that identify individual sequences, and could be targeted by combinations of probes, so that the efficient strategy of traditional taxonomic identification could be used in DNA diagnosis. The sizes of minimal sub-sequence sets depended mostly on sequence diversity and sub-sequence length and interactions between these parameters. We found that 201 distinct cytochrome oxidase subunit-1 (CO1) genes from moths (*Lepidoptera*) were distinguished using only 15 sub-sequences 20 nucleotides long, whereas only 8–10 sub-sequences 6–10 nucleotides long were required to distinguish the CO1 genes of 92 species from the 9 largest orders of insects.

**Conclusion:**

The presence/absence of sub-sequences in a set of gene sequences can be used like the questions in a traditional dichotomous taxonomic key; hybridisation probes complementary to such sub-sequences should provide a very efficient means for identifying individual species, subtypes or genotypes. Sequence diversity and sub-sequence length are the major factors that determine the numbers of distinguishing sub-sequences in any set of sequences.

## Background

In contemporary biological research, organisms are often identified by firstly sequencing one or more of their genes and then comparing the sequences with those of known species, either by inferring phylogenies or by database searches [1]. Once a sequence is available it may be used to design oligonucleotide probes, and these are used for most routine DNA diagnostic work, because probe hybridisation tests are far less expensive and less technically complex than sequence analysis. Specific oligonucleotide probes are used that are complementary in sequence to, and hence hybridise with, selected regions of the DNA, RNA or cDNA of the target species or genotype. Most such routine tests aim to identify specimens of a single species, or only a very few. Each probe is at least 18 nucleotides long and often twice as long, and is chosen so that it is unique and only hybridises with a single target. As a result, at least one specific probe is required for every target, although usually several different probes are used for each. In some tests, sets of species- or genotype-specific probes are deposited as arrays on solid supports, so that it is possible to check simultaneously if an unknown organism belongs to one or other of many different taxa or genotypes; this strategy is widely used for gene expression analysis. 'High density arrays' of such probes have been used on occasion for identifying pathogens [2], but they are not used routinely because, like sequence analysis, they are costly and technically complex [3], nonetheless the potential market for identifying pathogens in this way is very large (see Discussion).

By contrast, in traditional taxonomy, specimens are rarely identified using characters specific for an individual target, but, instead, by using combinations of characters shared by different members of a set of target organisms. In practice the characters are used to devise a series of presence/absence questions arranged as a 'dichotomous taxonomic key', so that answering these questions sequentially leads to the identification of a specimen. The main advantage of this strategy is that far fewer characters and questions are required to uniquely identify an individual target. The strategy is most efficient when each character bisects the targets into two equal groups, i.e. it is bisectionally distributed, and when different characters bisect the targets differently, ideally in a progressively binary way. In theory the minimum number of characters required to distinguish a finite number of targets by this method is defined by the binary logarithm X = log_2_Y, where X is the number of characters and Y is the number of targets. For example, ten ideal characters would, in theory, identify each of a set of 1024 targets, and only 20 ideal characters could identify more than a million targets; 1013 and 1,048,555 fewer tests respectively than using target-specific characters.

Using target-specific characters (i.e. one specific character for each target) is the least efficient strategy for identification when efficiency is measured as the number of characters required to identify a target. Using combinations of shared bisectionally-distributed characters can be much more efficient. The use of such shared characters is most efficient when they separate the targets in a progressively binary way.

In this paper we report that gene sequences contain sub-sequences that are present in quasi-randomly distributed sets of around half of the sequences, and hence their presence or absence could be used like the questions in a traditional taxonomic key. These sub-sequences could be detected by sets of probes with complementary sequences. A suitable set of such 'combinatorial probes' could be used to uniquely identify different individual DNAs as these would give different patterns of hybridisation, 'fingerprints', with different individual DNAs Sub-sequences that are suitable targets for probe combinations are most commonly 6–30 nts long. Sub-sequences of such lengths are not unique to the set of target genes, and so the target genes must first be separated from other 'contaminating' DNAs. Various physical or chemical techniques could be used to isolate the target sequences, but perhaps the most convenient would be by PCR using target region-specific primer mixtures (i.e. 'redundant' primers).

Attempts have been made to use algorithms based on suffix trees to find sub-sequences that could be used in combinations to distinguish between gene sequences [4], and others have used selection algorithms based on entropy maximisation [5,6] or on Lagrangian relaxation [6] to optimise probe selection. These studies focussed on the algorithmics of probe selection and demonstrated that sets of sub-sequences 5–8 nts long could distinguish individual sequences. Probes that are only 5 to 8 nts long are not widely used because they usually require unusual hybridisation conditions. In the work reported here we looked at a range of sub-sequence lengths and used a simple greedy algorithm, where sub-sequences were successively chosen that merely maximised the number of pairs of gene-sequences that were distinguished; the algorithm was based on suffix arrays because they use less computer memory than suffix trees to manipulate as large sets. Our study focussed on understanding the effect of gene-sequence diversity on the number and diversity of sub-sequences of different lengths that might be targeted by probes, as these factors will affect their use in practical applications. Here we report a study of three published sets of cytochrome oxidase c subunit 1 (CO-1) genes from representative groups of animal species [7,8]. These data were chosen because each set is consistent in length and composition, but differs greatly from the others in phylogenetic range and diversity. We have also studied, in less detail, several sets of sequences of plant and animal viruses and the ribosomal genes of bacteria.

## Results

Three sets of CO1 sequences were used; the details of which are available via the Internet [7,8]. The "CO1-animal" data was from 96 species of animals representing the seven dominant phyla of animals, the "CO1-insect" data was from 92 species of insects representing eight of the largest orders of insects and the "CO1-moth" data was from 201 species from three superfamilies of moths found near Guelph, Canada. After being aligned using ClustalX [9] with default parameters, the same region was selected from all three datasets for analysis; see Methods. The selected regions, the "test-sequences", were 604, 603 and 595 nucleotides long respectively in the three sets. Comparable random test-sequence datasets were constructed.

The three sets differed greatly in diversity: the CO1-animal set was the most diverse with an average nucleotide difference, ignoring positions that had gaps for alignment, between all pairs of sequences of 35.2% (S.D. 8.3%) with a range from 12.4% to 57%; the CO1-insect set had a mean nucleotide difference of 22.2% (S.D. 4.2%) with a range from 7.4% to 39.5%, and the CO1-moth set had a mean difference of 12.9% (S.D. 1.9%) with a range from 1.0% to 19.4%. Random sequence datasets were constructed that matched the length and average nucleotide composition of each test-sequence dataset, and had mean nucleotide differences of 72.1% to 73.3% (S.D. 1.8% to 1.9%). The CO1-animal sequences yielded a pool of 112,800 sub-sequences 6 nts long that included all replicates. Pools of sub-sequences up to 31 nts long were also produced and these were, of course, a little smaller. The CO1-insect sequences produced pools that were almost the same size, whereas those from the larger CO1-moth dataset were about twice as large.

### Distinguishing sub-sequences

It was assumed that two test sequences could be distinguished if one of them contained a sub-sequence and the other did not, even if the second contained a sub-sequence that differed from one in the first at only one position, as hybridisation methods to distinguish such sequences are well established for the assay of single nucleotide polymorphisms [10,11]. To find sub-sequences that could be used like questions in a taxonomic key we searched among those that were shared and bisectionally distributed. We excluded specific sub-sequences, namely sub-sequences that were singletons, and also all sub-sequences found in all the test sequence set. Therefore, the search was confined to "distinguishing sub-sequences" (DSSs), namely those that were present in at least two test-sequences but not present in all the test-sequences.

**Figure 1:**
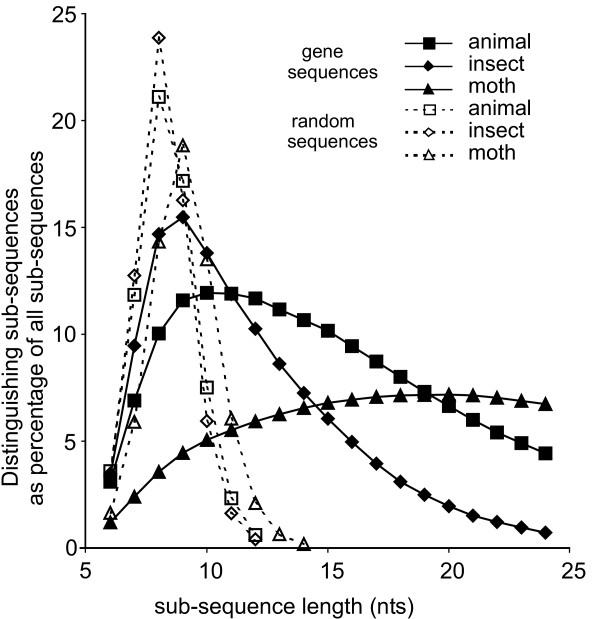
The percentage of DSSs of different lengths in the CO1 sequences, and in random sequences of the same length and composition.

Distinguishing sub-sequences (DSSs) constituted, at most, 15% of the sub-sequences in the pools from each CO1 sequence set (Fig. 1). Almost all nucleotide combinations up to 6 nucleotides (nts) long were present in all the sequences and, as they were uninformative and therefore eliminated, the percentage of DSSs tended towards zero for lengths less than 6 nts. The proportion of DSSs increased in pools of longer sub-sequences, but the number of singletons also increased with length, and so as sub-sequence length increased the percentage of DSSs in the sub-sequence pool peaked and then decreased. The position of the peak depended on sequence variation; the peak was found at lengths of 8 or 9 nts in pools from the random sequences, and at 9, 10 and 20 nts in those from the CO1-animal, CO1-insect and CO1-moth sequences respectively. Only a few short sub-sequences were repeated within any one sequence and so these had only a minor effect on the size of the DSS pool.

**Figure 2:**
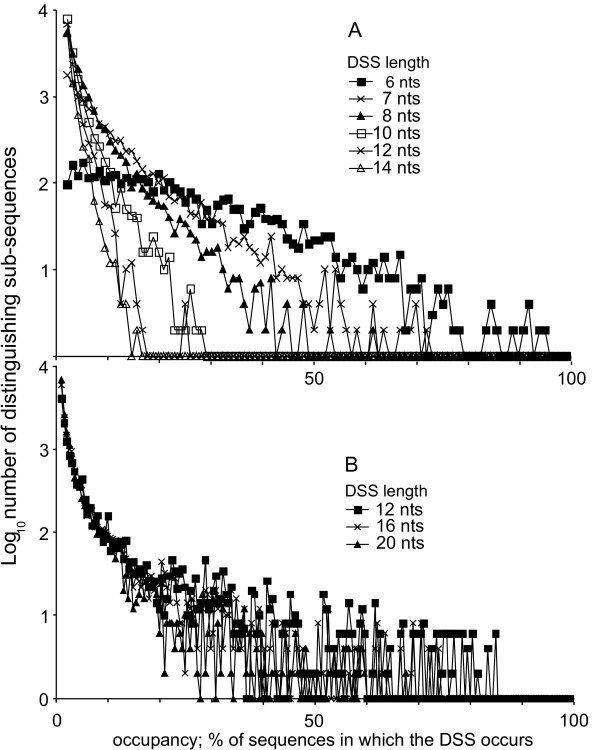
DSS occupancy; the number (log_10_) of DSSs of different lengths shared by different percentages of the test sequences in: A) the CO1-animal sequences; B) the CO1-moth sequences

Plots of the number of DSSs in each 'occupancy' category, namely the percentage of test-sequences in which each DSS occurred, showed large variations between the datasets (Fig. 2A &2B), and this mostly reflected the diversity of the sequences. Whereas the CO1-moth set yielded DSSs with 50% occupancy over the complete range of lengths tested, the CO1-insect sequences yielded none longer than 17 nts, and the CO1-animal sequences yielded none longer than 7 nts. In general, as the length increased so the number of DSSs in each occupancy category declined at approximately a negatively exponential rate, but there were large variations between the datasets. For all pools, most DSSs were present in fewer than 10% of the sequences and singletons were most common in pools of the longest DSSs, especially from the diverse CO1-animal data.

### Minimum complete sets (MC-sets) of CO1 sequences

Sets of DSSs that, in combination, would distinguish between test sequences were selected. A set of DSSs that could distinguish all the test-sequences in a dataset, in a manner like a taxonomic key, was considered a "complete set". A minimum complete set (MC-set) was defined as a set that contained the fewest DSS found by a random trajectory method (see Methods). Table 1 gives a MC-set for the CO1-moth sequences, and Table 2 gives the 'DSS signatures', binary barcodes or 'fingerprints' for some representative moths.

**Figure 3:**
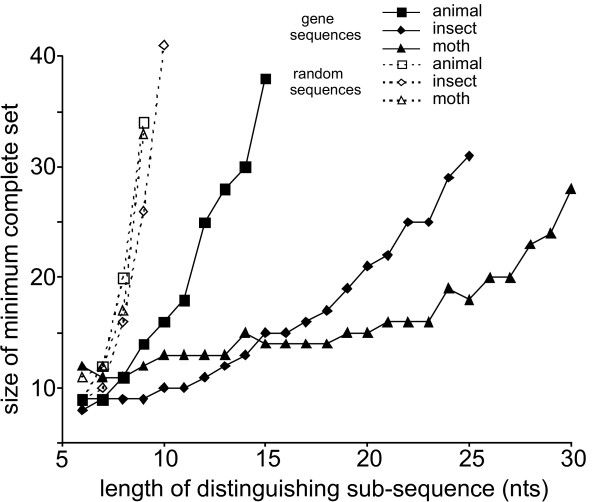
The minimum number of DSSs of different lengths that distinguish all sequences in each of the three CO1 datasets and in datasets of random sequences of the same length, number and average base composition.

MC-sets obtained from twenty searches each of the CO1-animal, CO1-insect and CO1-moth data consisted of only 9, 8 and 11 sub-sequences respectively (Fig. 3). In theory, 7 DSSs behaving in a perfectly dichotomous way would be required to distinguish all the sequences in the CO1-animal and CO1-insect data, and the CO1-moth data should require 8 DSSs. Thus, the MC-sets of the shortest DSSs were close to the theoretically predicted size. However, as DSS length and sequence diversity increased, so too did the sizes of the MC-sets. The increase was smoothly curvilinear with the random data, but more variable with real sequences. The more diverse sequences usually required larger MC-sets, although the greater size of the CO1-moth dataset also increased the MC-set size.

Each of the DSS pools was shown to contain several independent equally parsimonious MC-sets by successively excluding MC-sets from the pools and searching the depleted pools for new MC-sets. When this was done using the CO1-animal data and DSSs 8 nts long, the first MC-set was of 11 DSSs, but it was not until eight MC-sets had been successively removed that the MC-set size increased to 12. During the removal process the average occupancy of the DSSs in the MC-sets steadily declined from a mean of 40.2% (range 48% to 18%) to 29.7% (range 40% to 3%) When several MC-sets were obtained using the random DSS choice method and compared, it was clear that many DSSs from different MC-sets were interchangeable.

### Sub-sequence efficiency

**Figure 4:**
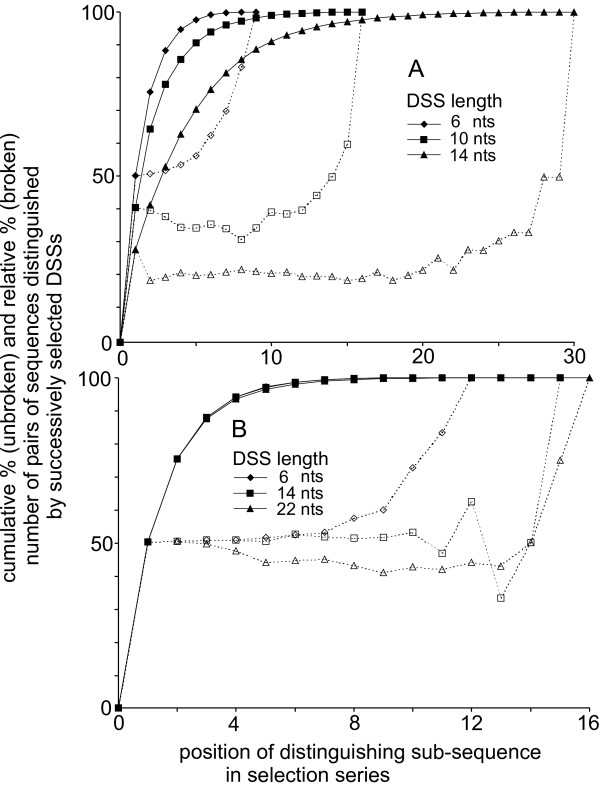
The cumulative and relative percentages of pairs of (A) the CO1-animal sequences and (B) the CO1-moth sequences, distinguished by successively selected DSSs. The 'relative efficiency' of each DSS is the number of pairs it distinguishes as a percentage of the pairs remaining to be distinguished.

The relative efficiency of each DSS within a complete set was assessed by calculating the percentage of sequence pairs it distinguished, from among those remaining to be distinguished when it was chosen. In this way, it was found that relative efficiency depended on whether suitable DSSs were available for selection, so whereas the first DSS selected from the CO1-animal sub-sequences 6 nts long was able to distinguish 50% of the sequences, the first DSSs that were 10 nts and 14 nts long only distinguished 41% and 28% of the sequences respectively (Fig. 4A &4B).

### Sequence groups

Our search method also allows groups of the sequences to be defined, so that the resulting MC-sets only contain DSSs that distinguished between members of different groups of sequences, but not necessarily between sequences of the same group. This enabled, for example, the 96 different CO1-animal sequences to be grouped into seven phyla (e.g. Chordata, Annelida, Nematoda, etc) but this only decreased the size of the MC-set for DSSs six nts long from 9 to 8 DSSs, and for DSS 12 nts long from 26 to 23. However grouping was more valuable for sequence sets containing many nearly identical variant sequences. For example a set of sequences from 240 isolates of *Potyvirus*, a genus of plant viruses, gave MC-sets of 22, 38 and 50 DSSs with sub-sequences 7, 10 and 12 nts long respectively, but when the sequences were grouped as the 62 recognized species the MC-sets were less than half the size; only 10, 14 and 19 DSSs respectively.

### Speed

The search method took 54 seconds to select an MC-set of 16 DSS 20 nts long from the 201 CO1-moth sequences when using one processor on a dual Opteron 242 processor machine running at 1.6 GHz. The same system took 13 seconds to select an MC-set of 17 DSSs 10 nts long from the 96 CO1-animal sequences. These tasks took 8 minutes 14 seconds and 85 seconds respectively in a PC with a Pentium CPU at 2.4 GHz. A version of the program is available for use for research purposes over the Internet, contact the corresponding author (MJG) for details.

## Discussion

All the studies described above in which the three sets of CO1 sequences were compared, illustrate the fact that the number of DSSs in a set of sequences is mostly determined by its diversity and by the length of the sub-sequences being sought. Short sub-sequences for probe targeting could readily be found, but longer sub-sequences that would be more useful for identification in standard hybridisation reactions were less common and more likely to be found among closely related, well conserved, gene sequences. The most useful sub-sequences for identification were, as predicted, those that were present in about half of the targets (i.e. those with occupancy scores of about 50%). Most gene sub-sequences less than 18 nts long are not unique to particular genes. Therefore they can only be used as targets for diagnostic tests when the target nucleic acids that contain them have been preselected in some way. This could be accomplished most conveniently by PCR using region-specific primers or primer mixtures.

One advantage of combining region-specific amplification with identification using combinatorial probes is that related but previously unrecognised or uncharacterised species or subtypes may be found. The chosen region, even from unknown species or subtypes, is likely to be amplified using the region-specific primers or primer mixture, and it is then also likely that the combinatorial probes will hybridise with at least some of the target sub-sequences, but will give DSS 'signatures' that have not been seen before. This is because each MC-set that we have found is many-fold redundant, and has the potential to generate many more different signatures than would be generated from the known test-sequences. For example, the MC-sets 18 nucleotides long that distinguished the 201 CO1-moth sequences were of 16 DSSs. Sixteen DSSs could, if they behaved in a perfectly dichotomous way, uniquely identify 65,536 different gene sequences or species (i.e. 2^16^). Thus the MC-sets we found were 99.7% redundant, and the combinations of DSSs not represented among the target sequences would be available to distinguish previously unknown variants of the selected gene region.

The aim of the work reported in this paper was to investigate the factors that influenced the numbers of sub-sequences that, in combination, could distinguish sequences or groups thereof. We therefore tested our selection algorithm using three published sets of CO1 sequences that were consistent in length and composition, but differed greatly from one another in phylogenetic range and diversity. We have also examined, but in less detail, a set of ribosomal RNA genes from 17 bacterial species representing 12 genera and also gene sequences from several groups of animal and plant viruses, namely flaviviruses, orthomyxoviruses, potyviruses and tobamoviruses (unpublished results). The results obtained with the bacterial and viral sequences did not differ in any significant way from those obtained with CO1 sequences, which suggests that there is no *a priori *reason to believe that DSSs for targeting by probe combinations are not present in all genes.

The design of practical diagnostic tests, based on the principles outlined in this paper, would involve several stages. First, known sequences of potential targets would be examined to find regions of convenient length and variability bracketed by conserved sites for PCR primers. The region-specific primers would be tested and optimised using a range of variant sequences. Then all known sequences of the region would be used to identify MC-sets of DSSs, whose complements could be used as probes in hybridisation-based tests to identify individual variants. However an iterative process will be required to design a working set of combinatorial probes as it is well known that a significant proportion of sub-sequences selected as hybridisation probes fail to behave as expected because of secondary structures in the target nucleic acid or the probe [12]. First an initial MC-set would be selected bioinformatically, then tested biochemically, and the probes that performed correctly used as a 'starter set' for further rounds of bioinformatic and biochemical selection, until a working MC-set was obtained. When this DSS set is used in practice, variant sequences giving unknown DSS signatures are likely to be found. These would then be sequenced and added to the trainer set, and the MC-set might have to be redesigned.

The value of target-specific 'high-density microarrays' of DNA probes was most spectacularly demonstrated when the pathogen causing SARS was shown to be a coronavirus. It was detected using an array of about 10,000 different oligonucleotides from some of the most conserved regions of about 1,000 reference viral genomes [2,13,14]. However, the microarrays used for SARS were not standard diagnostic tools, and high-density microarrays are also not used routinely in infectious disease diagnostics because of their cost and complexity [3]. Nonetheless multiplexing offers clear benefits [15] as more information is provided by each test.

At present non-multiplexed tests or tests that use just a few specific probes are the standard. These tests are used routinely for screening donor blood for viruses, including human immunodeficiency lentiviruses and hepatitis C hepaciviruses, and as the primary or confirmatory diagnostic tests for sexually transmitted pathogens and pathogens that cause meningitis [3,16-20]. These nucleic acid probe-based medical diagnostics have a very large market value [21].

Probes, which identify by being used in combinations, could be most usefully used in low-density DNA microarrays. Low-density microarrays typically comprise fewer than 100 probes and often fewer than 40 probes, and it seems likely that such microarrays could outperform high-density microarrays for routine diagnostic applications because of their reliability, simpler data analysis and much lesser cost [22-24]. Different combinatorial probe sets could be combined in each low-density array to achieve greater redundancy and accuracy; they might not merely replicate one another but could optimally target different major organism groups or different epidemiologically important strains with each replicate MC-set [19,25].

## Conclusion

This paper reports a method that finds sub-sequences which, in combinations, distinguish the individual gene sequences or groups of gene sequences from which they came, and that could be used as targets for DNA probes. Sequence diversity and sub-sequence length were found to be the major factors influencing the number of sub-sequences available as probe targets.

## Methods

### DATA

Three previously described datasets of CO1 sequences [7,8] were used, although certain sequences were not included either because they could not be retrieved from GenBank, or they were incomplete. The CO1-animal data was from 96 species of animals and lacks sequences AF310721, AJ271612, NC_002767 and AF370851 in the reported set; the CO1-insect data was from 92 species of insects and lacks sequences NC_003372, AY165779, AF146683, AB010925, NC_001566, NC_002084, NC_000857 and NC_001322, and the CO1-moth data was from 201 species of *Lepidoptera*. The sequences were aligned using Clustal X [9] with the default parameters, and the region providing the 'test sequences' was that bounded by the semi-conserved sequences 5'-GTNGGNACNGCNNT-3' and 5'-GGNGGNGGNGAYCC-3', which are potential gene specific PCR primer sites. Random sequence datasets were constructed that matched the length and average nucleotide composition of each test-sequence dataset.

Sets of sub-sequences that could distinguish the test-sequences were found using research programs written in Lahey Fortran 95. Test-sequences were degapped, and then every test-sequence in a dataset was initially converted into a pool of all the possible overlapping sub-sequences of a chosen length that it contained. Pools of sub-sequences of different lengths, ranging from 6 to 31 nucleotides (nts), were analysed separately. The uninformative sub-sequences that were discarded were singletons, replicates and sub-sequences found in all the test-sequences.

Sets of DSSs that, in combination, would distinguish between test sequences were selected by a "greedy algorithm". First, an array was constructed that recorded the DSSs in each test-sequence. A "distinguishing array" was then constructed that recorded for every pair of test-sequences, the DSSs that distinguished the pair. A "distinguishing-score" was then calculated for each DSS by summing the number of pairs of test-sequences that it distinguished. The DSS with the largest distinguishing-score was chosen. This DSS and the pairs that it distinguished were then eliminated from the distinguishing array. The process of DSS selection was then repeated either until a set of DSSs had been found that, in combination, distinguished all the test-sequences, or until no DSS could be found that would distinguish the remaining test-sequences. The set of DSSs that could distinguish all the test-sequences, was considered a "complete set". The ability of a complete set to distinguish the test-sequences in a dataset was independently confirmed by using a separate program to search the test-sequences for every DSS in the set, and by checking that the resulting pattern of its presence/absence, its "DSS signature", was unique.

During most searches, the greatest distinguishing score at each step of the search was achieved by more than one DSS, so one was chosen at random from among those with the greatest score at each step of the search for a complete set. This allowed a search to have a random trajectory through a succession of DSS choices, and often produced MC-sets of different sizes for the DSSs of the same length; the smallest were sometimes 3 DSSs smaller than the largest.

To aid the discovery of probe sets for different applications, options were included in the programs that permitted: (i) the exclusion of particular DSSs from the minimum set, (ii) inclusion of particular DSSs in the minimum set, (iii) exclusion of DSSs that, as double-stranded DNA, would 'melt' outside a chosen temperature range [26,27], and (iv) the exclusion of DSSs with runs of more than a defined number of consecutive residues of the same nucleotide.

## Authors' contributions

The authors contributed equally to this project. It was devised by MJG and AJG, all contributed equally to its development, JSA did all the programming, and AJG all the data testing.

## Figures and Tables

**Table 1:** A minimum complete (MC) set of DSSs that distinguish 201 CO1-moth sequences; the DSSs 18 nts long have predicted T_m_s in the range 37°–47°C and no consecutive 'runs' of more than three residues of the same nucleotide.

DSS	sequence
1	-ATAAAGGTATTTGATCAA-
2	-ATCCTCCAATTATAATAG-
3	-TCAAGAAGAATTGTAGAA-
4	-CTAATTCAGCTCGAATTA-
5	-TCATCTCCAATTAAAGAT-
6	-AAATTAATAGCTCCTAAA-
7	-GGAGGATTTGGAAATTGA-
8	-ATAAATTTGATCATCTCC-
9	-TCGAAATTTAAATACATC-
10	-GCAGGAACAGGATGAACA-
11	-TTTAGCTGGAGCTATTAC-
12	-AACAGATCGAAATTTAAA-
13	-ATTCGAGCAGAATTAGGA-
14	-AATTCTGCTCGAATTAGT-
15	-AAATGCAGTAATCCCTAC-
16	-AGAAGTATTTAAATTACG-

**Table 2:** Species representing various superfamilies of moths, together with the Accession Codes of their CO1 gene sequences and their 'DSS signatures', namely the presence/absence of the sub-sequences listed in Table 1 in the selected region of their CO1 gene sequences.

Species	Superfamily	Accession Code	DSS signature
*Ennomos subsignaria*;	Geometroidea	AF549628	1010110110110000
*Lobophora nivigerata*;	Geometroidea	AF549636	0001000000000000
*Lomographa vestaliata*;	Geometroidea	AF549637	1010000011001000
*Euchaetes egle*;	Noctuoidea	AF549609	1010000111011000
*Acronicta morula*;	Noctuoidea	AF549731	1000000001001000
*Hypena humuli*;	Noctuoidea	AF549743	0100010000000000
*Idia concise*;	Noctuoidea	AF549761	1101000001010000
*Orthosia alurina*;	Noctuoidea	AF549703	0111110011000000
*Orthosia hibisci*;	Noctuoidea	AF549725	0111100111000000
*Zale unilineata*;	Noctuoidea	AF549715	0111000000000000
*Gluphisia lintneri*;	Noctuoidea	AF549780	1100001101100001
*Ceratomia undulosa*;	Sphingiodea	AF549807	1100010110011000
*Smerinthus jamaicensis*;	Sphingiodea	AF549797	0010100100100000
*Sphecodina abbottii*;	Sphingiodea	AF549804	1100101010001000
